# A Novel Synthesis of Highly Efficient Antimicrobial Quaternary Ammonium Pyridine Resin and Its Application in Drinking Water Treatment

**DOI:** 10.3390/polym17131885

**Published:** 2025-07-07

**Authors:** Huaicheng Zhang, Haolin Liu, Wei Wang, Fengxia Dong, Yanting Zuo, Shouqiang Huang, Daqian Zhang, Ji Wu, Shi Cheng, Aimin Li

**Affiliations:** 1 School of Environment and Safety Engineering, Nanjing Polytechnic Institute, Nanjing 210044, China; huai.cheng@163.com (H.Z.);; 2 State Key Laboratory of Pollution Control and Resources Reuse, School of the Environment, Nanjing University, Nanjing 210023, China; liuhaolin@smail.nju.edu.cn (H.L.);; 3 School of Urban Construction, Changzhou University, Changzhou 213164, China; zuoyanting@cczu.edu.cn; 4 Jiangsu Key Laboratory of E-Waste Recycling, School of Resources and Environmental Engineering, Jiangsu University of Technology, Changzhou 213001, China; sqhuang@jsut.edu.cn (S.H.);; 5 State Key Laboratory of Hydrology-Water Resources and Hydraulic Engineering, Nanjing Hydraulic Research Institute, Nanjing 210029, China; wu1363516334@163.com

**Keywords:** quaternary ammonium polymer, antibacterial resin, optimal bactericidal alkyl, water disinfection, water purification

## Abstract

Multifunctional water-treatment materials urgently need to be developed to avoid normal organic matter, inorganic anions, resistant bacteria, and hazardous disinfection by-products in conventional drinking water treatment strategies. While quaternary ammonium pyridine resins (QAPRs) possess porous adsorption structures and incorporate antibacterial groups, enabling simultaneous water disinfection and purification, their limited bactericidal efficacy hinders broader utilization. Therefore, a deeper understanding of the structure-dependent antimicrobial mechanism in QAPRs is crucial for improving their antibacterial performance. Hexyl (C_6_) was proved to be the optimal antibacterial alkyl in the QAPRs. A new antibacterial quaternary ammonium pyridine resin Py-61 was prepared by more surficial bactericidal N^+^ groups and higher efficient antibacterial hexyl, performing with the excellent antibacterial efficiency of 99.995%, far higher than the traditional resin Py-6C (89.53%). The antibacterial resin Py-61 completed the disinfection of sand-filtered water independently to produce safe drinking water, removing the viable bacteria from 3600 to 17 CFU/mL, which meets the drinking water standard of China in GB5749-2022 (<100 CFU/mL). Meanwhile, the contaminants in sand-filtered water were obviously removed by the resin Py-61, including anions and dissolved organic matter (DOM). The resin Py-61 can be regenerated by 15% NaCl solution, and keeps the reused antibacterial efficiency of >99.97%. As an integrated disinfection–purification solution, the novel antibacterial resin presents a promising alternative for enhancing safety in drinking water treatment.

## 1. Introduction

Microbial contaminants in drinking water, particularly antibiotic-resistant bacteria (ARB) and disinfectant-resistant bacteria (DRB), are an increasingly critical problem threatening human health. Disinfection is an indispensable treatment process to prevent waterborne diseases by removing bacteria in the production of safe drinking water. Traditional disinfection agents, such as chlorine, chlorine dioxide, and ozone [1], inevitably generate hazardous disinfection byproducts (DBPs) and cause secondary pollution of residual disinfectants in drinking water disinfection, which results in hazards to people’s health [2,3]. Ultraviolet [4] radiation disinfection application is a promising disinfection technology, which forms fewer DBPs, but the bacterial regrowth via photoreactivation or dark repair of UV disinfection lowers its reliability in killing bacteria [5,6]. More critically, diverse resilient microbial strains have developed resistance to conventional disinfection methodologies [7,8,9], which increases the difficulty of drinking water disinfection.

Quaternary ammonium resins (QARs) are water-insoluble antibacterial polymers [10,11] that exhibit excellent performance in removing resistant bacteria and antibiotic-resistant genes (ARGs) in drinking water disinfection [12,13]. QARs achieve bacterial inactivation through electrostatic adhesion to anionic cell surfaces, and by disrupting bacterial membranes via hydrophobic alkyl chains. Unlike antibacterial materials relying on Ag^+^ ions [14,15] or reactive oxygen species (ROS) [16], this non-oxidative antimicrobial mechanism circumvents disinfectant resistance and DBPs formation associated with oxidative bactericidal processes. The inherent water insolubility and reusability of QARs facilitate their efficient separation and recovery from aqueous systems as compared with water-soluble traditional disinfectants, resulting in lower eco-toxicological effects [17,18,19] and lower water treatment costs. Although QARs have been reported for decades, with merits of killing bacteria, controlling ARGs, and good reusability, there are few practical applications in water environments on account of their limited improvements of bactericidal capacity. Quaternary ammonium pyridine resin (QAPR) is one important part of QARs which carries a nitrogen-containing pyridine ring that is a reported excellent bactericidal group [20,21], indicating the greater antibacterial improvement potentials of QAPR. As the reported structure-dependent antibacterial mechanism of quaternary ammonium acrylic resins (QAARs) [22], augmenting superficial N^+^ groups on QARs represents an effective strategy for enhancing antibacterial efficacy, and grafting the highly efficient antibacterial alkyl onto these surficial N^+^ groups. Referring to the antibacterial mechanism of QAARs, the antimicrobial mechanism of QAPRs urgently needs to be studied to develop a highly efficient antibacterial resin, aiming to meet water disinfection applications in water environment.

Here, this is first how to prepare an antibacterial QAPR with a rational design which could meet the disinfection and purification application in an actual water environment. Firstly, the optimal antibacterial alkyl in the quaternary ammonium pyridine resins (QAPRs) was proved to be hexyl (C_6_). Secondly, the brominated quaternary ammonium compound with hexyl (Br-QAC-C_6_) was synthesized successfully, which contains both the N^+^ group and the optimal antibacterial hexyl. The Br-QAC-C_6_ was grafted onto the surface of QAPR to simultaneously increase the quantities of surficial N^+^ groups and highly efficient bactericidal hexyl for higher antibacterial performance. Based on the former two findings, a novel antibacterial QAPR (Py-61) was synthesized via a rational two-stage quaternization protocol: (i) surface-selective functionalization with hexyl-bearing high-efficacy bactericidal quaternary ammonium compound (QAC-C_6_), and (ii) comprehensive bulk modification through grafting of methylated ammonium groups (C_1_) to maximize internal N^+^ density. The counter anions of Br^−^ and I^−^ in the resin Py-61 were exchanged into Cl^−^ by sodium chloride solution to reduce highly toxic DBPs (Br-DBPs or I-DBPs) [2]. Furthermore, the broad-spectrum bactericidal efficiency of Py-61 was conducted across multiple contamination scenarios, including simulated water and sand-filtered water, showing excellent antibacterial efficiency, far higher than other traditional bactericidal resins. The contaminant-removal capacity of resin Py-61 was also discussed in sand-filtered water, such as Br^−^, I^−^, HCO_3_^−^, SO_4_^2−^, total nitrogen (TN), total phosphorus (TP), and total organic carbon (TOC). In addition, the long-lasting reused antibacterial performance of the resin Py-61 was tested circularly with the used resin regenerated by sodium chloride solution. This work represents, to the best of our understanding, the initial exploration of introducing a highly efficient antibacterial quaternary ammonium compound (simultaneously carrying more surficial N^+^ groups and higher efficient bactericidal hexyl) covalently grafted onto the QAPR, and finishes the application research in actual water. This study provides a promising drinking-water treatment alternative for better drinking water quality and safety, which will markedly enhance the feasibility of implementing QAPR for integrated water treatment processes.

## 2. Materials and Methods

### 2.1. Materials

Commercial resins of D201 (a quaternary ammonium styrene-based resin) and D213 (a quaternary ammonium styrene-based resin) were purchased by Zhengguang Industrial Co., Ltd. in Hangzhou, China. Poly(4-vinylpyridine) resin (25% cross-linkage, 10–100 mesh) was bought from Sigma-Aldrich Company in Shanghai, China. Sodium tetraphenylborate analytical standard (ST) was purchased from Huaian Huake Chemical Co., Ltd. (Huai’an, China). Iodomethane, 1-iodobutane, 1-iodohexane 1-iodooctane, 1-iododecane, 1-iodododecane, methanol, absolute ethanol, acetone, acetonitrile, formaldehyde solution (33 wt.%), cetyltrimethy lammonium bromide (CTAB), titan yellow, and sodium hydroxide were all bought from J&K Company in Beijing, China. All the above reagents are of analytical grade. Bacteria of *S. aureus* (ATCC25923), *B. subtilis* (ATCC10719), *E. coli*, (ATCC8099), and *P. aeruginosa* (PAO1), were provided by the School of the Environment, Nanjing University, China.

### 2.2. Preparation of Antibacterial Poly(4-vinylpyridine) Resins

The antibacterial resins were prepared as follows [23]: First, 20 g of poly (4-vinylpyridine) resin (designated as Py-0, representing the non-quaternized base material), 60 mL of absolute ethanol, and 60 g of iodomethane were added into a 500 mL three-necked round-bottomed flask with a mechanical stirrer. The system was stirred continuously with 200 rpm at 38 °C, under reflux for 48 h. The obtained resins were extracted using acetone, methanol, and ethanol absolute, respectively. Finally, the resins were activated by sodium chloride solution (15 wt.%), washed 5 times with ultrapure water, and named Py-1C. Similarly, the antibacterial poly(4-vinylpyridine) resins of Py-4C, Py-6C, Py-8C, Py-10C, and Py-12C were prepared with 1-iodobutane, 1-iodohexane, 1-iodooctane, 1-iodododecane, and 1-iododecane, respectively.

The antibacterial resin of Py-61 was prepared as follows: Firstly, the N, N-dimethylhexylamine (0.15 moL) reacted with 1,3-dibromopropane (0.15 mol) at 76 °C under N_2_ atmosphere to produce the brominated quaternary ammonium compound with hexyl (Br-QAC-C6), as described by Zhang [24]. Secondly, the resin Py-0 (20 g) was superficially quaternized with Br-QAC-C6 (0.009 moL) added dropwise, and named Py-6N, referring to the above synthetic method. Thirdly, the obtained resins were fully quaternized with iodomethane as described before, and named Py-61-I. Finally, the resins were activated by sodium chloride solution (15 wt.%), and named Py-61. The synthetic and characteristic information of the above resins is recorded in Table S1.

### 2.3. Determination of Surficial N^+^ Charge Density

The surficial N^+^ charge density of the resins (surficial N^+^ groups of resins) is determined according to the method reported by Zhang [25]. In short, 100 mL of ultrapure water, 1 g of the resins, and 5 drops of sodium hydroxide (20 wt.%) were added to a 250 mL flask. Subsequently, the flask received sequential addition of (a) 10 mL of ST (0.02 mol/L), (b) 5 mL formaldehyde solution, and (c) 5 drops of 0.1 wt.% titan yellow indicator. The mixture underwent gentle agitation for 10 s followed by 40 min reaction maintenance at ambient conditions. The reaction was kept for 40 min. Finally, the excessive ST was titrated to the endpoint to show reddish-brown precipitates using the 0.01 mol/L CTAB solution [26]. The calculation formula was placed in Text S1.

### 2.4. Determination of Strong-Base Group Exchange Capacity

The strong-base group exchange capacity of resins (abbreviated as “exchange capacity”, i.e., total N^+^ groups of resins) was measured using the standard method 4.2 of GB/T11992-2008 [27]. In short, resins are added into a 10 mL exchange column, and then fully exchanged by the Na_2_SO_4_ solution (0.5 mol/L) to release the combined chloride ions (Cl^−^) from N^+^ groups. Finally, these released chloride ions from resins are quantified via the titration of silver nitrate solution (0.1 mol/L), with the potassium chromate (5% (*w*/*v*)) as an indicator. Similarly, the blank control group is titrated and recorded as V_1_. The calculation formula was placed in Text S2.

### 2.5. Antibacterial Testing

The bactericidal efficacy of QARs were quantified against two model Gram-negative bacteria (*P. aeruginosa* (PAO1) and *E. coli* (ATCC8099)), and two representatives of Gram-positive bacteria *B. subtilis* (ATCC10719) and *S. aureus* (ATCC25923) [28,29]. The four bacteria were cultured using Luria–Bertan [14] broth to achieve the optical density (OD) of approximately 0.20 at 600 nm. Bacterial suspensions were centrifuged, supernatants aspirated, and pellets resuspended in sterile ultrapure water. Final concentrations were normalized to approximately 10^5^ colony-forming units per milliliter (CFU/mL). In disinfection experiments, 100 mL of bacterial suspension and 1 mL of QARs were added to the flask, i.e., 100 bed volume (BV). Subsequently, the flasks were shaken at 200 rpm at 20 °C, for 40 min. Finally, the viable bacteria formed colonies after incubation at 37 °C for 24 h, and were counted via plate counting on the nutrient agar medium. The calculation formula is as follows:

(1) $$e\;=\;\frac{C0\;-\;C1}{C0}\;\times \;100\%$$

where *e* is the antibacterial efficiency (%); *C*0 and *C*1 are the viable bacteria in the blank control group and the experimental group, respectively (CFU/mL). To strengthen statistical validity, every antibacterial test group in this research underwent triplicate experimental runs. Resin reusability was assessed through sequential exposure cycles to fresh bacterial suspensions. Post-cycle regeneration involved immersion in 15% NaCl solution [22].

Statistical analysis and the characterizations of samples are in the Supplementary Materials, Text S3.

### 2.6. Statistical Analysis

The surficial N^+^ charge density, strong-base group exchange capacity, and antibacterial efficiency values, expressed in mean ± standard error, were calculated with Microsoft Excel 2016 (Microsoft, Redmond, WA, USA). Figures in this study were analyzed and obtained via Origin 8.5 (OriginLab, Northampton, MA, USA).

### 2.7. Analysis and Characterization

The particle sizes of resins were detected using a granulometer (Mastersizer 3000, Malvern, Worcester, UK). The specific surface areas and pore sizes of resins were determined based on the BET model (NOVA 3000e, Quantachrome, Boynton Beach, FL, USA). The FT-IR spectra of the samples were observed using a Fourier transform infrared spectrometer (NEXUS870, Thermo Fisher Scientific, Waltham, MA, USA). The optical densities (OD) of the bacterial liquids were recorded at a wavelength of 600 nm using a UV–vis spectrometer (UV-1800, Shimadzu, Kyoto, Japan). The total organic carbon (TOC) and total nitrogen (TN) of the samples were measureed using a TOC analyzer (Multi N/C 3100, Analytikjena, Jena, Germany). The selected experimental samples were investigated using a scanning electron microscopy (SEM) and energy dispersive spectrometry (EDS) (Quanta 250 FEG, FEI, Hillsboro, OR, USA).

## 3. Results and Discussion

### 3.1. Characterization of Poly(4-vinylpyridine) Resins

The FT-IR analyses of antibacterial QAPRs were conducted at the band range of 4000–500 cm^−1^. The FT-IR spectra (Figure S1) demonstrate that the tertiary amines in pyridine rings were quaternized into quaternary ammonium, with the vibration peak of C–N 1172 cm ^−1^, approaching that of the 1040 cm^−1^ reported by Zhang [24]. Moreover, a large amount of iodide ions (I^−^) were detected in the exchange capacity determination of QAPRs, which resulted from the resin quaternization reaction (one quaternary ammonium group (N^+^) combines with one iodide ion (I^−^)). The aforementioned results demonstrate that the tertiary amines in pyridine rings were quaternized by haloalkane (Iodoalkanes) successfully. Three skeleton resins possess the average pore sizes of 15–19 nm, determined on the basis of the BET model, and their particle sizes are 261–275 μm using a granulometer (in Table 1). These similar characteristic parameters ensure the accuracy of comparative analyses on antibacterial performances and contaminant removal capacities of different resins.

### 3.2. Optimal Antibacterial Alkyl Chain Length of Poly(4-vinylpyridine) Resins

**Figure 1:**
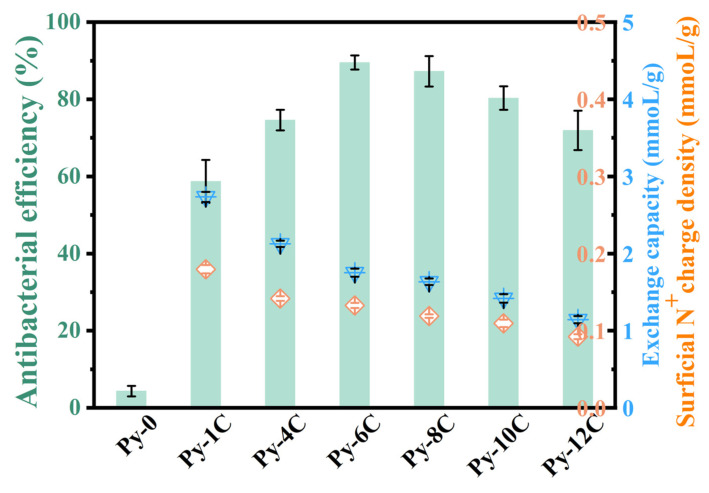
Effect of alkyl chain length of N^+^ groups of quaternary ammonium pyridine resins on antibacterial efficiency. Py-0: Non-quaternized poly(4-vinylpyridine) base resin. Py-nC: Quaternary ammonium resins synthesized via N-alkylation of Py-0 with iodomethane (C_1_, n = 1), 1-iodobutane (C_4_, n = 4), 1-iodohexane (C_6_, n = 6), 1-iodooctane (C_8_, n = 8), 1-iododecane (C_10_, n = 10), and 1-iodododecane (C_12_, n = 12). The error bars indicate the standard deviations from the obtained mean values. The bactericidal process employed 100 BV resin suspension agitated at 200 rpm, with isothermal maintenance at 20 °C throughout the 40 min treatment cycle.

Figure 1 shows that the quaternized poly(4-vinylpyridine) resins of Py-1C, Py-4C, Py-6C, Py-8C, Py-10C, and Py-12C all show significant antibacterial performance, different from the unquaternized resin Py-0 with insignificant bactericidal effect. The results prove that the quaternary ammonium groups are crucial for killing bacteria, and the pores in these resins cannot remove bacteria through physical adsorption, which is supported by Marques [23]. The surficial N^+^ charge density significantly decreases with increasing alkyl chain length, there is no significant difference between adjacent short chain resins (C_4_–C_6_ and C_8_–C_10_) (*p* > 0.05), and the chain length from C_6_ to C_8_ is a significant decrease in charge density. The exchange capacity significantly decreases with the increase in alkyl chain length, with no significant difference observed between C_6_ and C_8_ chain length resins (*p* > 0.05). The C_4_ chain length is the turning point for the significant decrease in capacity. Moreover, the optimal antibacterial alkyl of poly(4-vinylpyridine) resins was found to be hexyl (C_6_), exhibiting the highest bactericidal efficiency of 89.53%, similar to the reported poly(4-vinyl-N-alkylpyridinium bromide) material of Tiller [30]. The correlation between alkyl chain length and biocidal activity in QARs demonstrates peak efficacy at C_6_–C_10_ configurations, as substantiated by both prior research and our current study [31], which mediates alterations in polymeric interfacial behavior, topological states, and intermolecular recognition metrics [11], more efficient in damaging cell membranes and killing bacteria [32].

At the α = 0.05 level (ANOVA statistical analyses), there is a highly significant difference in the surficial N^+^ charge density (F (5, 12) = 587.60, *p* < 0.0001), exchange capacity (F (5, 12) = 367.50, *p* < 0.0001), and antibacterial efficiency (F (6, 14) = 205.68, *p* < 0.0001). The exchange capacity (total N^+^ groups) and surficial N^+^ charge density (surficial N^+^ groups) values of resins both decreased with their increasing antibacterial alkyl chain length. This is due to the higher steric hindrance of long-chain groups than those short-chain groups, which unavoidably lessens the total amount of reactions on the limited space of the resin. Consistent with our prior findings [22], extended alkyl chain length and elevated surface charge density of quaternary ammonium groups in acrylic resins (QAARs) demonstrably enhance antibacterial efficacy; however, these parameters exhibit an inherent trade-off (i.e., longer alkyl chains inevitably reduce surface N^+^ charge density). Consequently, the hexyl (C_6_) functionalization of poly(4-vinylpyridine) resins likely represents an optimal compromise between surface charge density and alkyl chain length, analogous to the reported maximally effective octyl (C_8_) moiety in QAARs [22]. Meanwhile, C_6_-modified poly(4-vinylpyridine) resins outperformed their C_1_ counterparts. This superiority, despite C_6_ resins possessing fewer N^+^ groups and thus reduced electrostatic attraction capacity, supports a primary contact-killing antibacterial mechanism (similar to other resins [25]), rather than bacterial removal via N^+^-mediated electrostatic adsorption. Furthermore, these findings indicate that within the tested alkyl chain lengths (<C_6_), long alkyl chains exert a stronger antibacterial influence than surficial N^+^ charge density, although both factors contribute positively to performance.

### 3.3. Rational Design and Preparation of Highly Efficient Antibacterial Poly(4-vinylpyridine) Resin Py-61

**Figure 2:**
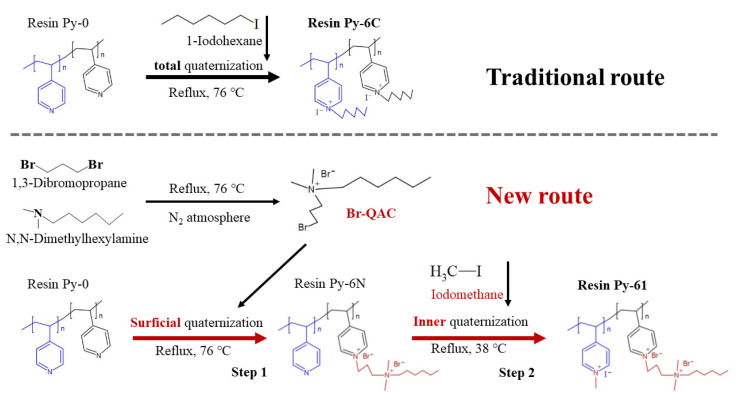
Traditional and new synthesis routes of antibacterial quaternary ammonium pyridine resin. Traditional routes for the preparation of the resin Py-6C, and new routes for the preparation of the resin Py-61, with two-step quaternization. The blue regions correspond to pyridyl moieties localized within the resin matrix core, whereas the black regions represent surface-bound pyridyl groups.

Based on the finding that the efficient bactericidal N^+^ groups in resins are mainly those surficial N^+^ groups quantified by the method of “surficial N^+^ charge density” [25], peak antimicrobial surface efficacy is attained through the strategic intensification of interfacial quaternary ammonium biocidal activity and by increasing the quantity of these groups. Combined with the optimal antibacterial hexyl (C_6_) of poly(4-vinylpyridine) resin, we designed a highly efficient antibacterial resin Py-61 via two-step quaternization (Figure 2): one with Br-QAC-C_6_ for grafting the QAC-C_6_ (for enhancing the antibacterial property of the surficial N^+^ groups), and the other with iodomethane for grafting the methyl C_1_ (for increasing the quantity of the inner N^+^ groups). From Figure S2, the Br-QAC-C_6_ and resin of Py-61 were demonstrated to complete their quaternization successfully, with the vibration peak of C–N 1172 cm^−1^. The exchange capacity and surficial N^+^ charge density values of the new resin Py-61 increase by 44.11% and 38.06% compared to that of the traditional antibacterial resin Py-6C (Table S1), which indicates the resin Py-61 has higher potentials in both removing contaminants and killing bacteria.

In Figure S3, the surficial N^+^ group quantity of Py-6N approaches its total N^+^ group quantity (95.03%), which indicates that the first quaternization reaction mainly occurs on the surface of resins. Furthermore, the increasing total N^+^ group quantity of Py-6N is far higher than its increasing surficial N^+^ group quantity (243 times), which indicates that the second quaternization reaction mainly occurs inside the resins and generates abundant interior N^+^ groups. Moreover, the surficial N^+^ groups (grafting with C_6_) in the resin Py-6N are 88.74% of those (grafting with C_6_ and C_1_) in the resin Py-61-I, and the inner N^+^ groups (grafting with C_6_) in the resin Py-6N are 0.94% of those (grafting with C_6_ and C_1_) in the resin Py-61-I. These results demonstrate that most surficial N^+^ groups are grafted with C_6_, and most inner N^+^ groups are grafted with C_1_.

**Figure 3:**
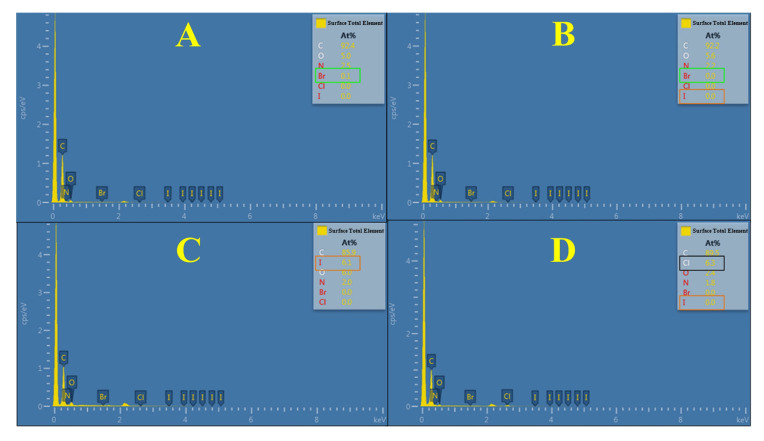
EDS analysis of different quaternary ammonium pyridine resins. (**A**) Halogen elements were analyzed through scanning the surface of the resin Py-6N by energy dispersive spectrometry (EDS); (**B**) Halogen elements were analyzed through scanning the interior of the resin Py-6N by EDS; (**C**) Halogen elements were analyzed through scanning the interior of the resin Py-61-I by EDS; (**D**) Halogen elements were analyzed through scanning the interior of the resin Py-61 by EDS, and the counter anions of the resin Py-61were exchanged into chlorine ions by sodium chloride solution.

In addition, a small amount of bromine emerged on the resin surface in Figure 3A, not in the resin interior in Figure 3B, which shows that the first quaternization of resin Py-61 using Br-QAC-C_6_ mainly occurs on the surface of resins. This is because bromine only appears at the resin quaternization reaction sites involving Br-C bonds. Similarly, a large amount of iodine emerged in Figure 3C, not in Figure 3B, showing that the second quaternization of resin Py-61 using iodomethane mainly occurs in the interior of resins. From Figure 3D, only chloride ions (Cl^−^) were detected in the resin Py-61, which indicates that the counter anions of I^−^ in the resin Py-61-I (in Figure 3C) could be exchanged into Cl^−^ by sodium chloride solution. In conclusion, a highly efficient antibacterial resin Py-61 was rationally designed and prepared via two-step quaternization successfully. The new resin Py-61 completed the first and second quaternization on its surface and interior, respectively, with a higher surficial N^+^ charge density and exchange capacity, which confer its better potentials in antibacterial performance and contaminant-removal capacity than that in the traditional antibacterial resins.

### 3.4. Antibacterial Performance of Resin Py-61 in Simulated Water

**Figure 4:**
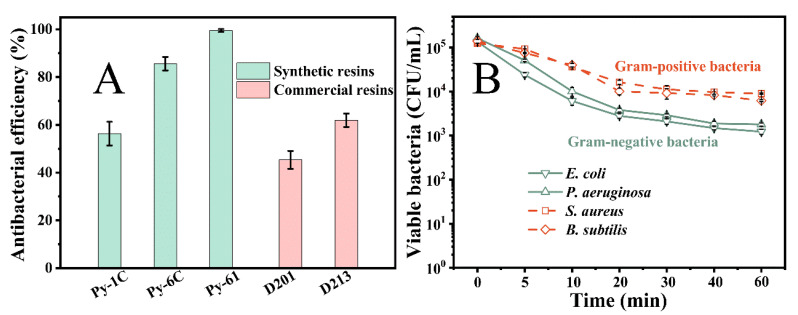
High-efficiency and broad-spectrum antibacterial performance of Py-61 resin in simulated water. (**A**) Antibacterial performances of different quaternary ammonium resins; (**B**) broad-spectrum antibacterial performance of the resin Py-61. The bactericidal process employed 100 BV resin suspension agitated at 200 rpm, with isothermal maintenance at 20 °C throughout the 40 min treatment cycle.

Figure 4A indicates that the new resin Py-61 has an excellent antibacterial efficiency of 99.995%, higher than the traditional resin Py-6C (85.61%), and superior to the commercial resins D201 and D213 (*p* < 0.05). As mentioned previously, this is attributed to the higher surficial N^+^ charge density (i.e., more surficial antibacterial N^+^ groups) and better bactericidal hexyl (C_6_) in the resin Py-61. Figure 4B shows that the resin Py-61 performs good broad-spectrum antibacterial properties on the Gram-positive bacteria of *S. aureus* and *B. subtilis*, and Gram-negative bacteria of *E. coli* and *P. aeruginosa*. Furthermore, the resin Py-61 exhibits more efficiency in eliminating the Gram-negative bacteria than the Gram-positive bacteria. This outcome is attributable to the thinner cell walls of Gram-negative bacteria (compared to Gram-positive counterparts) [33], facilitating easier permeation and subsequent inactivation by antibacterial N^+^ groups. This conclusion was supported by Rashed’s research on the antibacterial dental composite [34], which had more advantages in killing *P. aeruginosa* than *S. aureus*. These results prove that our ingenious bactericidal structure design of resin Py-61 is sufficiently effective, providing a workable preparation route for antibacterial resin.

Regarding reusability, the novel resin Py-61 exhibited a gradual decline over the initial 10 cycles, stabilizing thereafter with minor fluctuations around 99.97% (Figure S4). This sustained performance demonstrates its reliable reusability and cost-effectiveness.

### 3.5. Disinfection and Purification Performance of Resin Py-61 in Sand-Filtered Water

**Figure 5:**
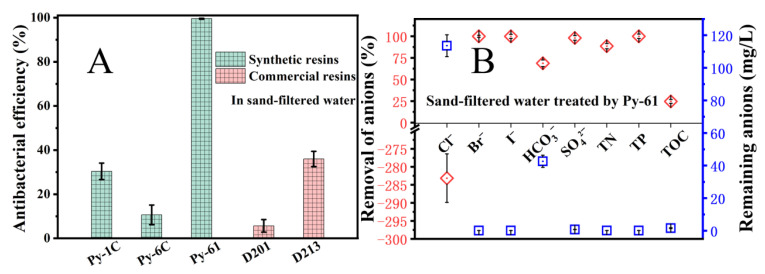
Disinfection and purification performance of resin Py-61 in sand-filtered water. (**A**) Disinfection performance of different quaternary ammonium resins; (**B**) purification performance of the resin Py-61. The bactericidal process employed 100 BV resin suspension agitated at 200 rpm, with isothermal maintenance at 20 °C throughout the 40 min treatment cycle.

The disinfection and purification performances of the resin Py-61 were assessed using sand-filtered water, with its characteristic parameters summarized in Table S1. Figure 5A shows that the new resin Py-61 has the highest antibacterial efficiency, better than the resins of Py-1C, Py-6C, D201, and D213 in sand-filtered water, similar to the results in simulated water. The antibacterial resin Py-61 completed the disinfection of sand-filtered water independently to produce safe drinking water, removing the viable bacteria from 3600 to 17 CFU/mL, which meet the drinking water standard of China in GB5749-2022 [35] (<100 CFU/mL). However, the antibacterial performance of Py-6C is lower than that of Py-1C, D201, and D213, different from the conclusion in simulated water.

The cationic N^+^ moieties within the resin matrix electrostatically attract anionic species including Cl^−^, HCO_3_^−^, and SO_4_^2−^, as well as negatively charged macromolecules (e.g., TOC components) and bacterial cell surfaces. When antibacterial N^+^ sites engage with competing anions or organic adsorbates, their bactericidal efficacy is compromised due to reduced accessibility to bacterial targets. Consequently, the interference resistance of antimicrobial resin is governed by the surface density of accessible bactericidal N^+^ groups. The result shows the resin Py-6C carries less surficial antibacterial N^+^ groups than other resins (Table 1), making it easier to be interfered with by the negatively charged contaminants in sand-filtered water. This finding demonstrates that the resin Py-61 with a higher surficial N^+^ charge density and exchange capacity has a better anti-interference property and bactericidal adaptability in actual water than the traditional antibacterial resin Py-6C. Simultaneously, Figure 5B shows that the contaminants in sand-filtered water were obviously removed, such as Br^−^ (100%), I^−^ (100%), HCO_3_^−^ (68.84%), SO_4_^2−^ (98.16%), TN (88.68%), TP (100%), and TOC (24.67%), similar to the F-Elements removal by quaternary pyridine resin [36]. These removed contaminants would significantly improve drinking water quality, greatly decrease the demand and cost for coupled chlorine disinfectant, and subsequently reduce the production of DBPs [28,37].

While maintaining bactericidal efficacy equivalent to silver-incorporated polymer composites, Py-61 demonstrates substantially lower production expenditures due to its ligand-free metal synthetic pathway [38]. Exhibiting a 75% reduction in contact time relative to carbon-doped g-C3N4 microtubes (120 min), Py-61 accomplishes 99% bacterial inactivation within 30 min, demonstrating superior kinetic efficiency [39]. The photocatalytic performance of nanocomposite materials is constrained by the accelerated recombination kinetics of photogenerated electron–hole pairs, with concurrent alterations in aqueous-phase constituents (e.g., pH, ionic strength) further attenuating quantum efficiency through competitive photon absorption/scavenging mechanisms [40,41]. Py-61 demonstrates significant resilience against common ions while efficiently eliminating common inorganic ions from water, resulting in improved water quality.

In summary, the new resin Py-61 could meet the disinfection and purification application in an actual water environment, offering a viable option for drinking water treatment. This integrated disinfection and purification system aims to enhance water quality and safety.

## 4. Conclusions

In summary, the optimal antibacterial alkyl of the QAPRs was proved to be hexyl (C_6_). The Br-QAC-C_6_ was synthesized successfully to simultaneously increase the quantities of surficial N^+^ groups and highly efficient bactericidal hexyl for higher antibacterial performance of QAPRs. Based on the former two findings, a new antibacterial poly(4-vinylpyridine) resin Py-61 was prepared with a higher surficial N^+^ charge density and exchange capacity, showing an excellent antibacterial efficiency of 99.995%, far higher than the traditional resin Py-6C (89.53%). The antibacterial resin Py-61 completed the disinfection of sand-filtered water independently to produce safe drinking water and meet the limit of drinking water (GB5749-2022). Meanwhile, the contaminants in sand-filtered water were obviously removed by the resin Py-61, such as Br^−^ (100%), I^−^ (100%), HCO_3_^−^ (68.84%), SO_4_^2−^ (98.16%), TN (88.68%), TP (100%), and TOC (24.67%). The counter anions of Br^–^ and I^–^ in the resin Py-61 could be exchanged into Cl^−^ by sodium chloride solution to reduce highly toxic DBPs (Br-DBPs or I-DBPs). The resin Py-61 can be regenerated by 15% NaCl solution, and maintains the reused antibacterial efficiency of >99.97%. Given these advantages, antibacterial methods present a promising avenue for drinking water treatment. Specifically, their ability to integrate disinfection and purification functions holds potential for improved water quality and safety. Future progress in this field is expected to significantly advance the deployment of QAPRs for water purification and disinfection purposes.

## Figures and Tables

**Table 1:** Characteristic information of quaternary ammonium resins.

Resins	Alkyl Chain Length	Cross Linkage	Size (μm)	Average Pore Size (nm)	Strong-Base Group Exchange Capacity (mmol/g)	Surficial N^+^ Charge Density (mmol/g)
Py-0	None	25%	50~80 Mesh/275 μm	16.06	None	None
Py-1C	C_1_/methyl	25%	50~80 Mesh/275 μm	16.01	2.7350	0.1797 ± 2.94%
Py-4C	C_4_/butyl	25%	50~80 Mesh/275 μm	16.01	2.1264	0.1418 ± 2.18%
Py-6C	C_6_/hexyl	25%	50~80 Mesh/275 μm	15.42	1.7522	0.1327 ± 2.53%
Py-8C	C_8_/ octyl	25%	50~80 Mesh/275 μm	15.39	1.6350	0.1191 ± 1.93%
Py-10C	C_10_/ decyl	25%	50~80 Mesh/275 μm	15.27	1.4190	0.1095 ± 4.29%
Py-12C	C_12_/dodecyl	25%	50~80 Mesh/275 μm	15.24	1.1435	0.0923 ± 3.57%
Py-61	C_6_ + C_1_	25%	50~80 Mesh/275 μm	15.87	2.5251	0.1832 ± 2.19%
D201	C_1_/methyl	20%	50~80 Mesh/261 μm	17.45	3.5107	0.1811 ± 1.62%
D213	C_1_/methyl	20%	50~80 Mesh/270 μm	18.26	3.4435	0.1753 ± 1.55%

## Data Availability

The original contributions presented in this study are included in the article/Supplementary Materials. Further inquiries can be directed to the corresponding authors.

## Supplementary Materials

The following supporting information can be downloaded at: https://www.mdpi.com/article/10.3390/polym17131885/s1, Text S1: The calculation formula for surficial N+ Charge density; Text S2: The calculation formula for strong-base group exchange capacity; Text S3: Analysis and characterization; Figure S1: FT-IR spectra of quaternary ammonium pyridine resins; Figure S2: FT-IR spectra of different resins and chemical compounds; Figure S3: N^+^ groups distribution of the resin Py-61 on the two-step quaternization; Figure S4: Reused antibacterial performance of the resin Py-61 in ten cycles; Table S1: Sand-filtered water characteristic parameters; Table S2: ANOVA statistical analyses for mean surficial N^+^ charge density; Table S3: Tukey grouping for mean surficial N^+^ charge density; Table S4: Tukey grouping results for mean surficial N^+^ charge density; Table S5: ANOVA statistical analyses for exchange capacity; Table S6: Tukey grouping for exchange capacity; Table S7: Tukey grouping results for exchange capacity; Table S8: ANOVA statistical analyses for antibacterial efficiency; Table S9: Tukey grouping for antibacterial efficiency; Table S10: Tukey grouping results for antibacterial efficiency; Table S11: ANOVA statistical analyses for bactericidal rate; Table S12: Tukey grouping for bactericidal rate; Table S13: Tukey grouping results for bactericidal rate; Table S14: ANOVA statistical analyses for mean bactericidal rate in sand filtered water; Table S15: Tukey grouping for mean bactericidal rate in sand filtered water; Table S16: Tukey grouping results for mean bactericidal rate in sand filtered water.
