# Supplementary material for: A Novel Synthesis of Highly Efficient Antimicrobial Quaternary Ammonium Pyridine Resin and Its Application in Drinking Water Treatment
Source: Polymers (Basel). 2025 Jul 7;17(13):1885. doi: 10.3390/polym17131885 (PMC12252215; doi:10.3390/polym17131885)
Supplement: Supplementary file 1 [file polymers-17-01885-s001.zip › polymers-3645263-supplementary.pdf]

Supporting Information for

“A Novel Synthesis of Highly Efficient Antimicrobial Quaternary Ammonium Pyridine Resin and its Application in Drinking Water Treatment”

Huaicheng Zhang[1,2], Haolin Liu[2], Wei Wang[1], Fengxia Dong[1], Yanting Zuo[3], Shouqiang Huang[4], Daqian Zhang[4], Ji Wu[5], Shi Cheng[4,*], Aimin Li[2]

1 School of Environment and Safety Engineering, Nanjing Polytechnic Institute, Nanjing 210044, China

2 State Key Laboratory of Pollution Control and Resources Reuse, School of the Environment, Nanjing University, Nanjing 210023, China

3 School of Urban Construction, Changzhou University, Changzhou 213164, China

4 Jiangsu Key Laboratory of E-waste Recycling, School of Resources and Environmental Engineering, Jiangsu University of Technology, Changzhou 213001, China

5 State Key Laboratory of Hydrology-Water Resources and Hydraulic Engineering, Nanjing Hydraulic Research Institute, Nanjing, 210029, China

[*]**Corresponding author**

Email: shicheng@jsut.edu.cn

Submitted to *Polymers*

CONTENTS

1. **Text**

    Text S1 The calculation formula for surficial N^+ Charge density
    Text S2 The calculation formula for strong-base group exchange capacity

    Text S3 Analysis and characterization

2. **Figures**

    Figure S1 FT-IR spectra of quaternary ammonium pyridine resins

    Figure S2. FT-IR spectra of different resins and chemical compounds

    Figure S3. N^+ groups distribution of the resin Py-61 on the two-step quaternization

    Figure S4. Reused antibacterial performance of the resin Py-61 in ten cycles

3. **Tables**

    Table S1 Sand-filtered water characteristic parameters

    Table S2 ANOVA statistical analyses for mean surficial N^+charge density

    Table S3 Tukey grouping for mean surficial N^+ charge density

Table S4 Tukey grouping results for mean surficial N^+charge density

Table S5 ANOVA statistical analyses for exchange capacity

Table S6 Tukey grouping for exchange capacity

Table S7 Tukey grouping results for exchange capacity

Table S8 ANOVA statistical analyses for antibacterial efficiency

Table S9 Tukey grouping for antibacterial efficiency

Table S10 Tukey grouping results for antibacterial efficiency

Table S11 ANOVA statistical analyses for bactericidal rate

Table S12 Tukey grouping for bactericidal rate

Table S13 Tukey grouping results for bactericidal rate

Table S14 ANOVA statistical analyses for mean bactericidal rate in sand-filtered water

Table S15 Tukey grouping for mean bactericidal rate in sand-filtered water

Table S16 Tukey grouping results for mean bactericidal rate in sand-filtered water

Text

Text S1 The calculation formula for surficial N$^+$ Charge density

$$\sigma = \frac{(C_1 \times V_1 - C_2 \times V_2)}{m \times (1 - X)} \quad (1)$$

Where σ, the surficial N$^+$ charge density (mmol/g); C_1 and C_2, the concentrations of ST and CATB solutions, respectively (mol/L); V_1 and V_2, the volumes of ST and CATB solutions, respectively; m, resin weight (g); X, the water retention capacity of resins (%).

Text S2 The calculation formula for strong-base group exchange capacity

$$Q = \frac{(V2 - V1)C1}{m(1 - w)} \quad (2)$$

Where Q, the strong-base group exchange capacity (mmol/g); C1, the concentration of silver nitrate solution (mol/L); V1 and V2, the titration volume of the blank control group and test sample, respectively [26]; m, the test sample weight (g); *w*, the water retention capacity of test sample (%).

Text S3 Analysis and characterization

The antibacterial efficiency, exchange capacity, and surficial N$^+$ charge density detection value, expressed as the mean ± standard error, were calculated by Microsoft Excel 2016 (Microsoft, USA). The experimental data were analyzed and visualized using Origin 8.5 (OriginLab, USA) and SPSS 22.0 (IBM SPSS Statistics).

FT-IR characterization of the QARs was conducted on a Thermo Scientific™ NEXUS870 spectrometer (USA). The UV-vis spectrometer (UV-1800; Shimadzu, Japan) was used to determine the optical density (OD) of bacterial liquids at the wavelength of 600 nm. The total organic carbon and total nitrogen of water samples were measured using the TOC analyzer (Multi N/C 3100; Analytikjena, Germany). Specific surface area and pore size distribution of resin matrices were characterized using the BET theory implemented on a NOVA 3000e surface area analyzer (Quantachrome Instruments, USA). Morphological and elemental analyses were performed via field-emission scanning electron microscopy (FE-SEM; Quanta 250 FEG) coupled with energy-dispersive X-ray spectroscopy (EDS) (Thermo Fisher Scientific, USA).

Figures

Figure S1 FT-IR spectra of quaternary ammonium pyridine resins.

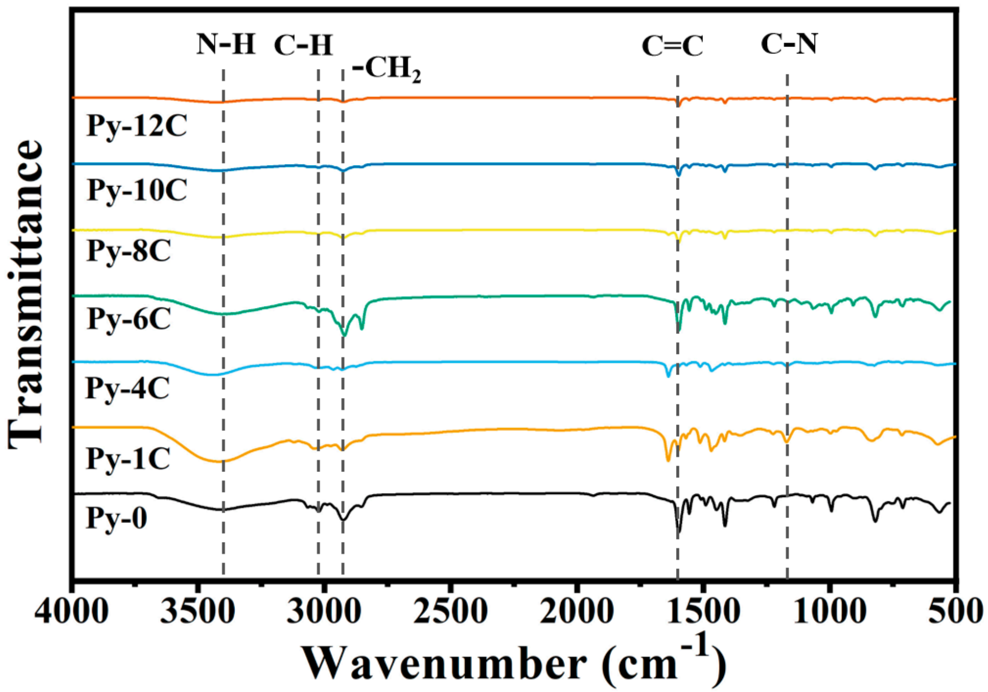

The resins of Py-1C, Py-4C, Py-6C, Py-8C, Py-10C, and Py-12C were synthesized by quaternization with different alkyl-chain iodoalkanes. The vibration peaks of C–N 1172 cm^{-1} indicate that the tertiary amines in pyridine rings were quaternized into quaternary ammonium. The

antibacterial alkyls in the resins of Py-1C, Py-4C, Py-6C, Py-8C, Py-10C, and Py-12C are methyl, butyl, hexyl, octyl, decyl, and dodecyl, respectively. Disinfection experimental conditions: resin dosage of 100 BV (bed volume), rotation speed of 200 rpm, at 20°C for 40 min.

Figure S2. FT-IR spectra of different resins and chemical compounds

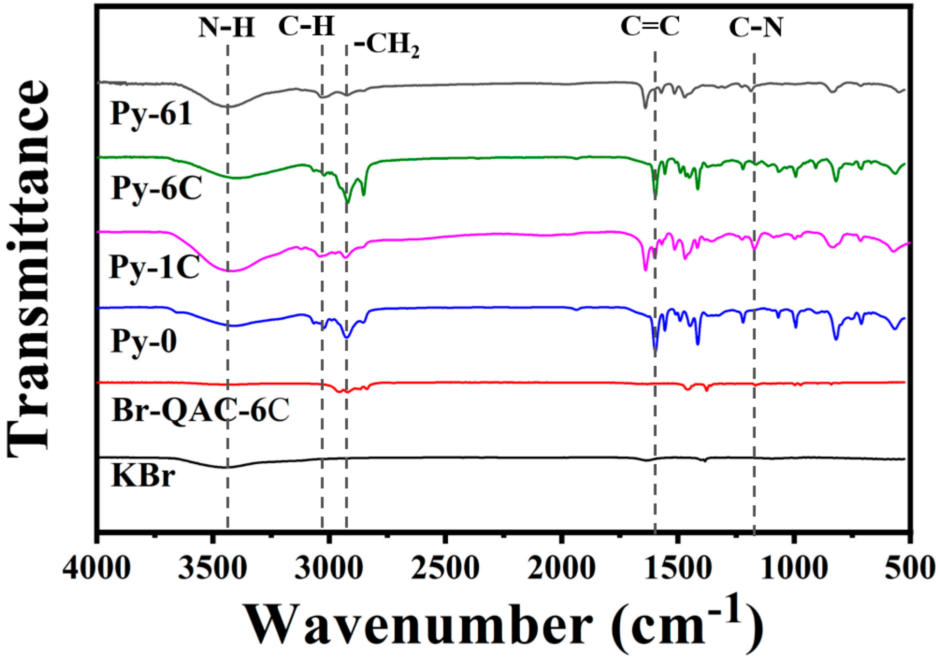

KBr: the chemical reagent of potassium bromide. Br-QAC-C_6: the brominated quaternary ammonium compound containing the antibacterial hexyl was synthesized, as described in the Materials and Methods in article. Py-0: The resin is the original quaternary ammonium pyridine resin with no quaternization. Py-1C and Py-6C: the resin Py-0 was quaternized by iodomethane and 1-iodohexane, respectively. Py-61: the resin Py-0 was quaternized by Br-QAC and iodomethane in sequence, and activated by 15% (wt. %) NaCl solution.

Figure S3. N+ groups distribution of the resin Py-61 on the two-step quaternization

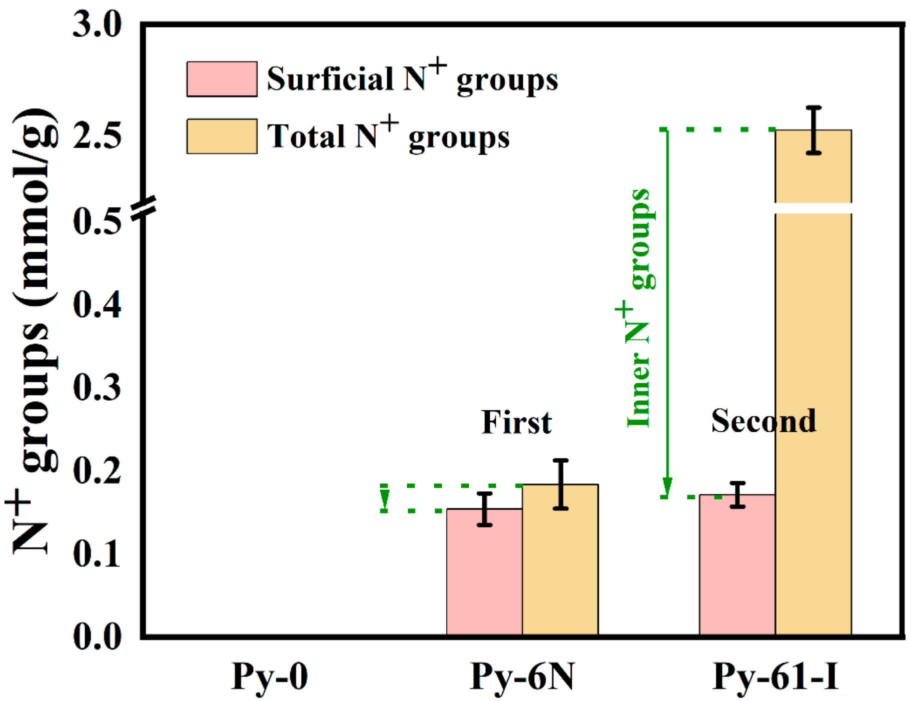

Py-0: The resin is the original quaternary ammonium pyridine resin with no quaternization. Py-6N: the resin Py-0 was quaternized by the synthetic compounds f Br-QAC-C_6. Py-61-I: the resin Py-0 was quaternized by Br-QAC and iodomethane in sequence. In the two-step quaternization, the first quaternization reaction mainly occurred on the surface of resins and generated the surficial N^+ groups (grafting with QAC-C_6), and the second quaternization reaction mainly occurred in the interior of resins and generated the inner N^+ groups (grafting with C_1).

Figure S4. Reused antibacterial performance of the resin Py-61 in ten cycles

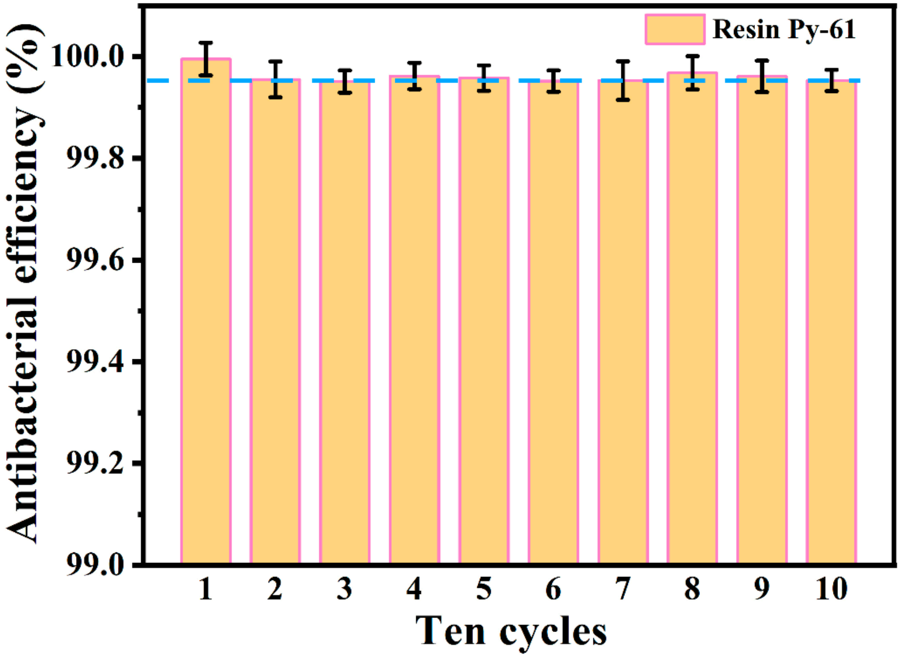

Reusability of the resin Py-61 was evaluated via adding resins into a fresh bacteria suspension to initiate sequential experiments. The used resins were regenerated with 15% sodium chloride (NaCl) solution at each cycle.

Tables

Table S1 Sand-filtered water characteristic parameters

water	Cl^- (mg/L)	Br- (μg/L)	I^- (μg/L)	HCO_3^- (mg/L)	SO_4^{2-} (mg/L)	TN (mg/L)	TP (mg/L)	NOM (mg/L)	Viable bacteria (CFU/mL)
FW	29.63	10.52	6.67	136.57	37.04	0.53	0.14	1.98	3600
FW+Py-61	113.52	N/A	N/A	42.55	0.68	0.06	N/A	1.49	17

Table S2 ANOVA statistical analyses for mean surficial N^+ charge density

Source of variation	Free degree(df)	Square sum (SS)	Mean square (MS)	F-value	p-value
Between groups	5	0.04267	0.00711	587.60	< 0.0001
Within groups	12	0.00017	0.000012		
Total	17	0.04284			

Table S3 Tukey grouping for mean surficial N^+ charge density

Comparison	Mean difference(%)	95% confidence interval	p-value	significance
Py-0 vs Py-1C	-0.17970	[-0.186, -0.173]	<0.0001	***
Py-0 vs Py-4C	-0.14180	[-0.148, -0.136]	<0.0001	***
Py-1C vs Py-12C	+0.08740	[+0.081, +0.094]	<0.0001	***
Py-4C vs Py-12C	+0.04950	[+0.043, +0.056]	<0.0001	***
Py-1C vs Py-10C	+0.07020	[+0.064, +0.077]	<0.0001	***
Py-6C vs Py-12C	+0.04040	[+0.034, +0.047]	<0.0001	***
Py-1C vs Py-8C	+0.06060	[+0.054, +0.067]	<0.0001	***
Py-4C vs Py-10C	+0.03230	[+0.026, +0.039]	<0.0001	***

Table S4 Tukey grouping results for mean surficial N^+ charge density

Resin	Mean Surficial N^+ Charge Density	Tukey Group
Py-1C	0.17970	**a**
Py-4C	0.14180	**b**
Py-6C	0.13270	**b**
Py-8C	0.11910	**c**
Py-10C	0.10950	**c**
Py-12C	0.09230	**d**

Table S5 ANOVA statistical analyses for exchange capacity

Source of variation	Free degree(df)	Square sum (SS)	Mean square (MS)	F-value	p-value
Between groups	5	4.7595	0.9519	367.50	< 0.0001
Within groups	12	0.0311	0.0026		
Total	17	4.7906			

Table S6 Tukey grouping for exchange capacity

Source of variation	Free degree(df)	Square sum (SS)	Mean square (MS)	F-value
Py-1C vs Py-12C	+1.5915	[+1.452, +1.731]	<0.0001	***
Py-1C vs Py-10C	+1.3160	[+1.176, +1.456]	<0.0001	***
Py-1C vs Py-8C	+1.1000	[+0.960, +1.240]	<0.0001	***
Py-1C vs Py-6C	+0.9828	[+0.843, +1.123]	<0.0001	***
Py-1C vs Py-4C	+0.6086	[+0.469, +0.748]	<0.0001	***
Py-4C vs Py-12C	+0.9829	[+0.843, +1.123]	<0.0001	***
Py-4C vs Py-10C	+0.7074	[+0.568, +0.847]	<0.0001	***
Py-6C vs Py-12C	+0.6087	[+0.469, +0.748]	<0.0001	***
Py-8C vs Py-12C	+0.4915	[+0.352, +0.631]	<0.0001	***
Py-6C vs Py-8C	-0.1172	[-0.257, +0.023]	0.142	ns

Table S7 Tukey grouping results for exchange capacity

**Resin**	**Mean Exchange Capacity**	**Tukey Group**
Py-1C	2.7350	a
Py-4C	2.1264	**b**
Py-6C	1.7522	**c**
Py-8C	1.6350	**c**
Py-10C	1.4190	**d**
Py-12C	1.1435	**e**

Table S8 ANOVA statistical analyses for antibacterial efficiency

Source of variation	Degrees of freedom(df)	Square sum (SS)	Mean square (MS)	F-value	p-value
Between groups	6	15511.83	2585.305	205.68	< 0.0001
Within groups	14	175.97	12.5696		
Total	20	15687.80			

Table S9 Tukey grouping for antibacterial efficiency

**Source of variation**	**Mean difference**	**F-value**	**Source of variation**	**Mean difference**	**F-value**
**Py-1C vs Py-12C**	1.5915	***	**Py-4C vs Py-12C**	0.9829	***
**Py-1C vs Py-10C**	1.3160	***	**Py-4C vs Py-10C**	0.7074	***
**Py-1C vs Py-8C**	1.1000	***	**Py-4C vs Py-8C**	0.4914	***
**Py-1C vs Py-6C**	0.9828	***	**Py-4C vs Py-6C**	0.3742	***
**Py-1C vs Py-4C**	0.6086	***	**Py-6C vs Py-12C**	0.6087	***
**Py-6C vs Py-10C**	0.3332	***	**Py-8C vs Py-12C**	0.4915	***
**Py-6C vs Py-8C**	0.1172	ns	**Py-8C vs Py-10C**	0.2160	***
**Py-10C vs Py-12C**	0.2755	***	**Py-8C vs Py-6C**	0.1172	ns

Table S10 Tukey grouping results for antibacterial efficiency

**Resin**	**Mean Efficiency**	**Tukey Group**
Py-6C	89.53	**a**
Py-8C	87.24	**a**
Py-10C	80.31	**A b**
Py-4C	74.61	**b**
Py-12C	71.92	**b**
Py-1C	58.72	**c**
Py-0	4.34	**d**

Table S11 ANOVA statistical analyses for bactericidal rate

**Source of variation**	**Degrees of freedom(df)**	**Square sum (SS)**	**Mean square (MS)**	**F-value**	**p-value**
Between groups	4	4392.617	1098.154	126.459	< 0.0001
Within groups	10	86.842	8.684		
Total	14	4479.459			

Table S12 Tukey grouping for bactericidal rate

Comparison	Mean difference(%)	95% confidence interval	p-value	significance
Py-61 vs D201	+54.658	[49.13, 60.19]	<0.0001	***
Py-61 vs Py-1C	+43.650	[38.12, 49.18]	<0.0001	***
Py-61 vs D213	+38.083	[32.55, 43.61]	<0.0001	***
Py-6C vs D201	+40.276	[34.75, 45.80]	<0.0001	***
Py-6C vs Py-1C	+29.268	[23.74, 34.80]	<0.0001	***
Py-6C vs D213	+23.701	[18.18, 29.23]	<0.0001	***
D213 vs D201	+16.575	[11.05, 22.10]	0.0003	**
Py-1C vs D201	+11.008	[5.48, 16.53]	0.0008	**

Table S13 Tukey grouping results for bactericidal rate

Resin	Mean Bactericidal Rate (%)	Tukey Group
Py-61	99.995	**a**
Py-6C	85.613	**b**
D213	61.912	**c**
Py-1C	56.345	c
D201	45.337	**d**

Table S14 ANOVA statistical analyses for mean bactericidal rate in sand-filtered water

Source of variation	Free degree(df)	Square sum (SS)	Mean square (MS)	F	p
difference between groups	4	28047.982	7011.995	584.332	< 0.0001
intra group differences	10	120.000	12.000		
total variance	14	28167.982			

Table S15 Tukey grouping for mean bactericidal rate in sand-filtered water

Comparison	Mean difference(%)	95% confidence interval	p-value	significance
Py-61 vs D201	+93.880	[+89.86, +97.90]	<0.0001	***
Py-61 vs Py-6C	+88.900	[+84.88, +92.92]	<0.0001	***
Py-61 vs Py-1C	+69.180	[+65.16, +73.20]	<0.0001	***
Py-61 vs D213	+63.570	[+59.55, +67.59]	<0.0001	***
D213 vs Py-6C	+25.330	[+21.31, +29.35]	<0.0001	***
D213 vs D201	+30.310	[+26.29, +34.33]	<0.0001	***
Py-1C vs Py-6C	+19.720	[+15.70, +23.74]	<0.0001	***
Py-1C vs D201	+24.700	[+20.68, +28.72]	<0.0001	***
D213 vs Py-1C	-5.610	[-9.63, -1.59]	0.0032	**
Py-6C vs D201	+4.980	[+0.96, +9.00]	0.0126	*

Table S16 Tukey grouping results for mean bactericidal rate in sand-filtered water

Resin	Mean Bactericidal Rate (%)	Tukey Group
Py-61	99.53	**a**
D213	35.96	b
Py-1C	30.35	**b**
Py-6C	10.63	**c**
D201	5.65	**c**
